# Supplementary material for: Combination Therapies Targeting Apoptosis in Paediatric AML: Understanding the Molecular Mechanisms of AML Treatments Using Phosphoproteomics
Source: Int J Mol Sci. 2023 Mar 16;24(6):5717. doi: 10.3390/ijms24065717 (PMC10058112; doi:10.3390/ijms24065717)
Supplement: Supplementary file 1 [file ijms-24-05717-s001.zip › ijms-2211266-supplementary.pdf]

Supplementary Figure Legends

S1: Cytotoxicity testing of each of the single compounds from the 10 hit well. Compounds were tested over a period of 72 h at a final concentration of 0.1 μM and 0.5 μM. Cells were treated with 0.1 % DMSO as a vehicle control. Cell toxicity was assessed at 72 h using CellTox Green Cytotoxicity Assay which was added to the cell suspension and cells were incubated for 20 minutes at room temperature before reading. Relative fluorescence unit (RFU) (Ex: 485 nm, Em: 520 nm) was measured using a Synergy HTX Multi- mode Microplate reader. Data is representive of N=3 ±SEM. * = $p < 0.05$. ** = $p < 0.01$, **** = $p < 0.0001$.

S2: Dose response curves to determine IC50 values to optimize the ideal dosing for the double and triple combinations. Cell viability was determined using CellTiter® Glo following 72 h. Luminescence was measured using a Synergy HTX Multi- mode Microplate reader. Data is representive of N=3 ±SEM.

S3: Corresponding densometric quantification of PARP, cleaved-PARP, procaspase-9, cleaved caspase-9, procaspase-3, and cleaved caspase-3 in Kasumi-1 and MV4-11 cell lines following 72 h treatment following 72 h treatment as single agents and as a combination. GAPDH was used as a loading control. Data is representive of representive of N=3 ±SEM.
