# Supplementary figures and images for: Combination Therapies Targeting Apoptosis in Paediatric AML: Understanding the Molecular Mechanisms of AML Treatments Using Phosphoproteomics
Source: Int J Mol Sci. 2023 Mar 16;24(6):5717. doi: 10.3390/ijms24065717 (PMC10058112; doi:10.3390/ijms24065717)

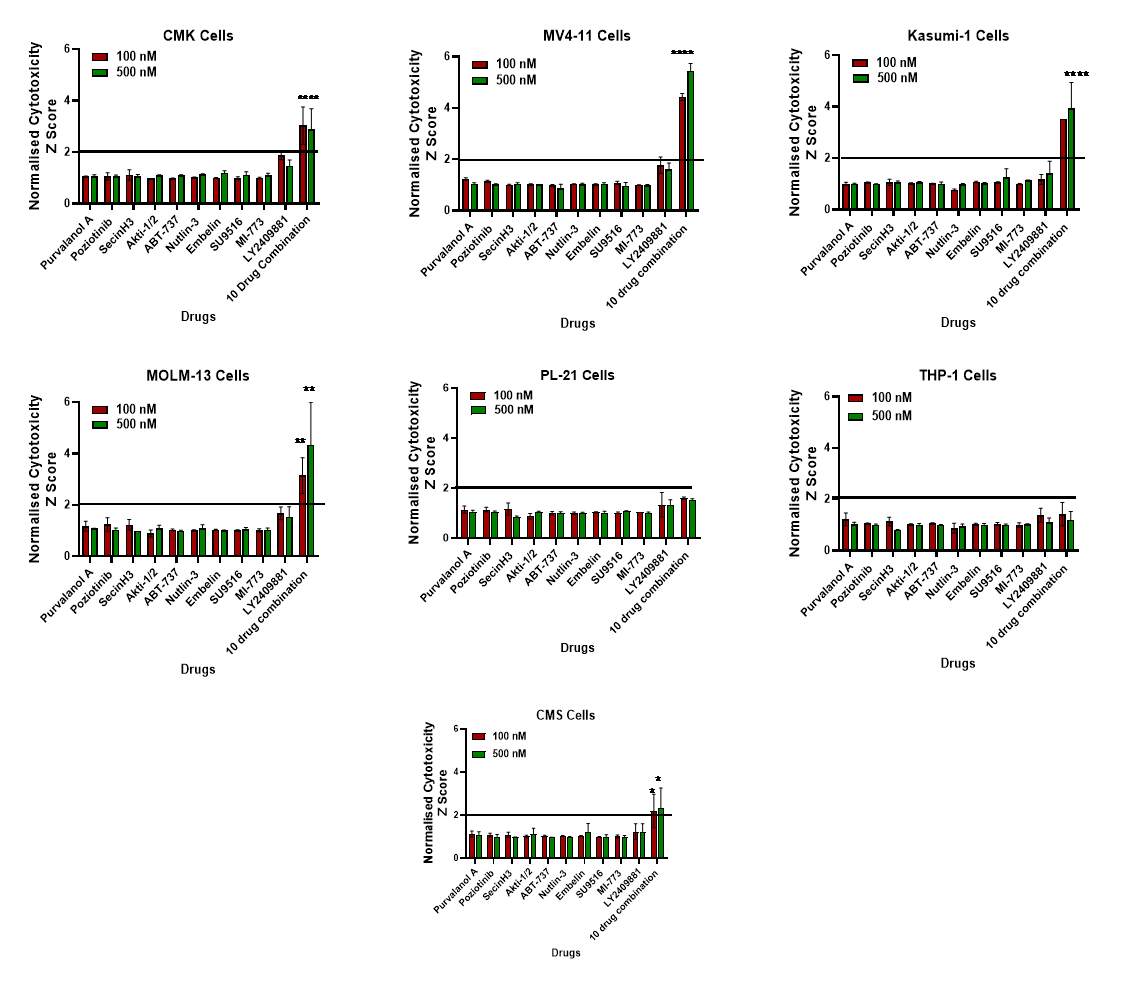

Supplement: Supplementary file 1 [file ijms-24-05717-s001.zip › Supplementary Figure S1 .tif]

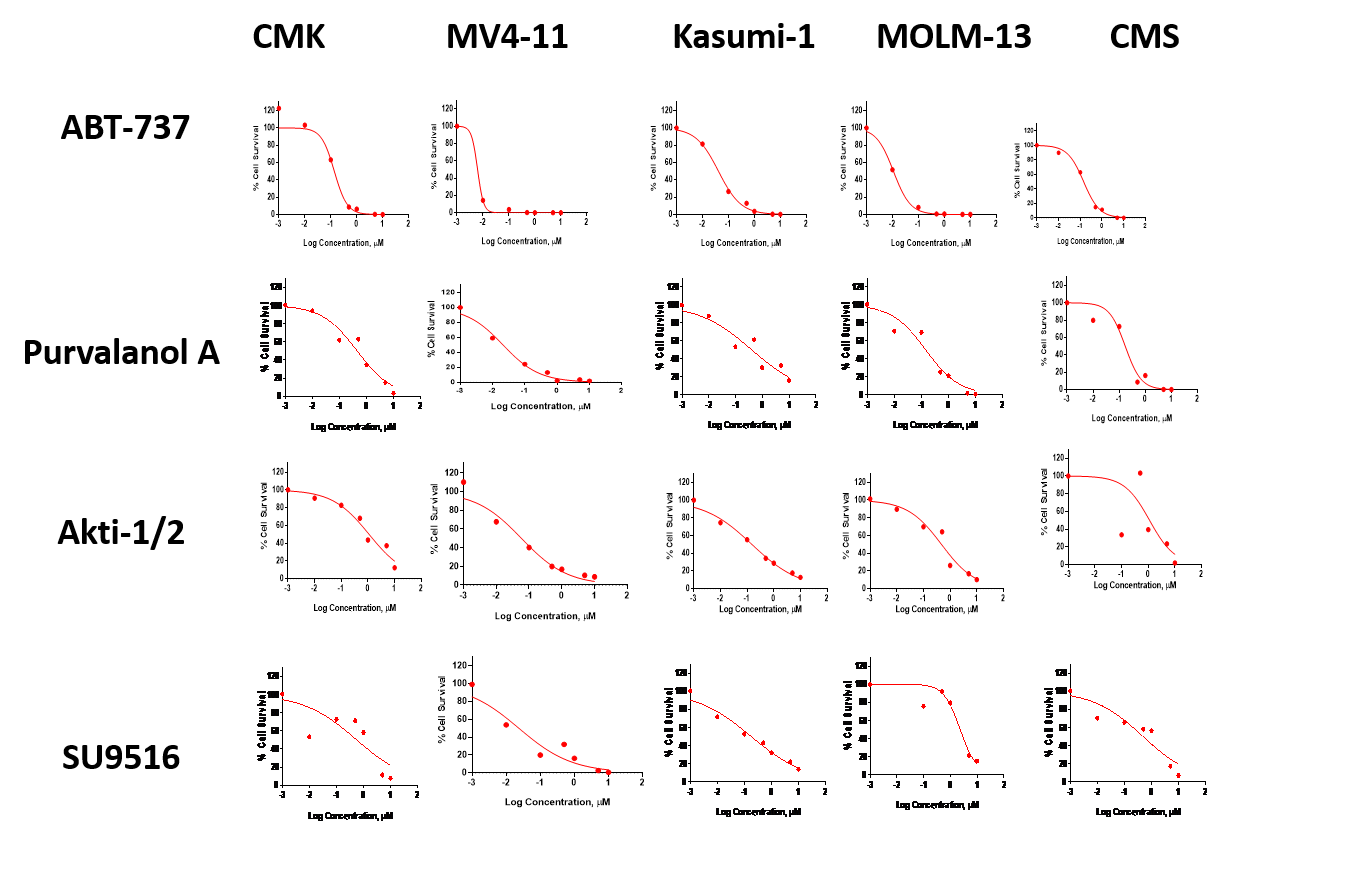

Supplement: Supplementary file 1 [file ijms-24-05717-s001.zip › Supplementary Figure S2.tif]

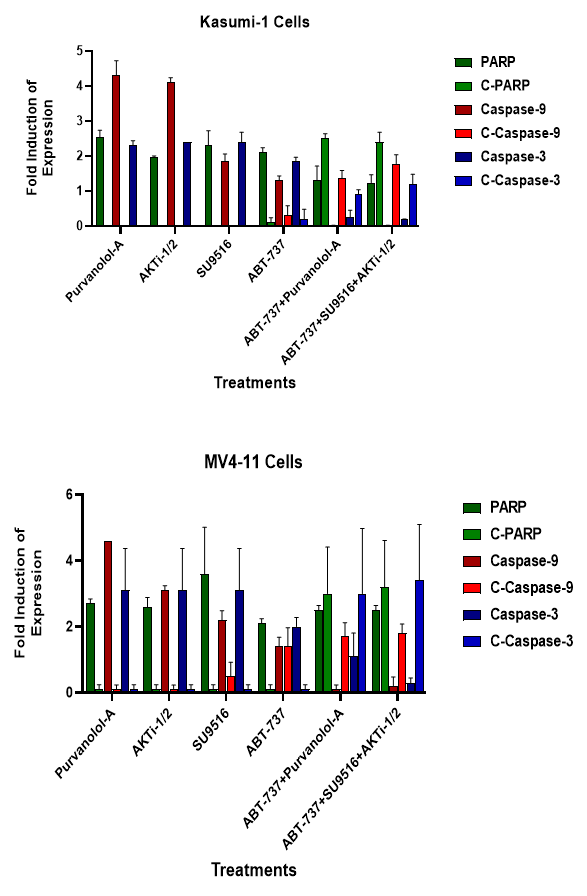

Supplement: Supplementary file 1 [file ijms-24-05717-s001.zip › Supplementary Figure S3.tif]
